# The Role of Connexin in Ophthalmic Neovascularization and the Interaction between Connexin and Proangiogenic Factors

**DOI:** 10.1155/2022/8105229

**Published:** 2022-06-22

**Authors:** Chuyang Xu, Hong Zhang, Wei Zhong, Hongyan Zhou

**Affiliations:** Department of Ophthalmology, China–Japan Union Hospital of Jilin University, Changchun, China

## Abstract

The formation of new blood vessels is an important physiological process that occurs during development. When the body is injured, new blood vessel formation helps the body recuperate by supplying more oxygen and nutrients. However, this mechanism can have a negative effect. In ophthalmologic diseases, such as corneal new blood vessels, neonatal vascular glaucoma, and diabetes retinopathy, the formation of new blood vessels has become a critical component in patient survival. Connexin is a protein that regulates the cellular and molecular material carried by cells. It has been demonstrated that it is widely expressed in vascular endothelial cells, where it forms a slit connection between adjacent cells to promote cell-cell communication via hemichannels, as well as substance exchange into intracellular environments. Numerous studies have demonstrated that connexin in vascular endothelial cells plays an important role in angiogenesis and vascular leakage. The purpose of this study was to investigate the effect between the angiogenesis-associated factor and the connexin. It also reveals the effect of connexin on ophthalmic neovascularization.

## 1. Introduction

Blood vessels can deliver nutrients to the entire body under physiological conditions, promoting body growth and wound healing and ameliorating ischemic tissue damage. Pro- and antiangiogenic factors regulate angiogenesis. When the balance is disrupted, numerous pathogenic alterations can occur. Angiogenesis exacerbates the difficulty of establishing successful treatment in proangiogenesis pathological conditions, including ophthalmic diseases [1]. In healthy condition, cornea is transparent tissue and it is an important refractive tissue to maintain vision. After trauma or inflammation, blood vessels grow into the transparent cornea from the limbus, endangering vision [2]. Severe diabetic retinopathy and wet age-related maculopathy, both of which are caused by abnormal vascular growth and leakage into the retina, eventually result in retinal hemorrhage, severe ischemia, and hypoxia, impairing vision [3]. There are two types of neovascularization: neovascularization from the original vascular network (sprouting) and nonsprouting angiogenesis, also referred to as intussusceptive angiogenesis [4, 5]. In the transitional period, pathological blood vessels sprout and differentiate into neovascularization and subsequently become immature blood vessels, which lack a complete vascular structure and are prone to leakage. Following that, immature blood vessels differentiate into tip cells and ultimately mature into blood vessels [6]. Angiogenic factors are overexpressed during pathological vascular formation. Numerous inflammatory cells infiltrate and release a variety of factors, including VEGF, TNF-*α*, IL-1*β*, PDGF, and EGF [7]. These angiogenic factors have an effect on angiogenesis in the surrounding microenvironment, and researchers attempt to inhibit this process by blocking the transmission of these factors.

A critical component of maintaining body homeostasis is maintaining the stability of signal communication from cell to cell or from cell to the external environment [8]. In comparison to other cell contact protein complexes, connexin plays a unique role. Every six proteins form a hemichannel, and the hemichannel between adjacent cells forms a gap junction. The gap junction allows the movement of a variety of substances with molecular weights up to 1000 Da between cells. Along with intercellular communication, these hemichannel proteins enable the cell to exchange substances with its surrounding microenvironment [8–10]. There are 21 connexin genes in human tissues, including Cx43, Cx36, Cx45, Cx32, and Cx57. Connexins can self-assemble into homopolymers and heteropolymers. The vascular system expresses gap junction proteins Cx40, Cx43, Cx37, Cx45, and Cx32, respectively, of which Cx43 is the most abundant, and hence the protein has been the most researched [11, 12]. The CT terminal of Cx43 is an important sequence because it can regulate a variety of signaling pathways. This is referred to as the noncanonical connexin route. It is precise because the CT terminal of Cx43 is rich in proline and serine that it serves as a target for the interaction of numerous proteins and kinases, hence regulating cell function. Cx43 CT has been demonstrated to influence the mitotic signaling pathway, hence controlling cell growth, differentiation, and migration [13].

Environmental stimuli have a significant influence on angiogenesis, and these stimuli can affect the function and expression of connexin in vascular endothelial cells. Interestingly, altering connexin expression in vascular endothelial cells can also alter their function, thus affecting angiogenesis [14, 15]. The purpose of this study is to collect existing evidence regarding the effect of relevant factors on connexin expression during angiogenesis. Simultaneously, we discovered how changes in connexin affect ophthalmic neovascular diseases.

## 2. Effect of Connexin on Vascular Development

In the Cx43 knockout mouse model, the expressions of angiogenesis-related genes such as the VEGF signal pathway gene VEGFA and NOTCH signal pathway genes Jagged1, DLL4, and Notch4 were found to be overexpressed in the heart tissue. Increased expression of these genes resulted in abnormal coronary artery branches in mice, impairing their heart development. The mice died early and had a decrease in distal coronary artery branches, indicating a vascular remodeling defect. The findings indicate that Cx43 is required for the formation and remodeling of coronary vessels [16]. Similarly, in the Cx43 knockout mouse, defects in the early stages of cardiac primitive coronary plexus remodeling were seen [17, 18].

Cx43-deficient mice exhibited a similar effect on hepatic vein angiogenesis. After establishing a mouse cholestasis model, it was discovered that while heterozygous knockout mice had normal bile duct development, hepatic venous angiogenesis was significantly decreased [19]. Diabetes-induced downregulation of Cx43 expression increases diabetic retinopathy, a retinal vascular disease. At the same time, the researchers discovered that Cx43 heterozygous knockout mice had similar vascular alterations, most notably loss of retinal pericytes and an increase in acellular capillaries. It is hypothesized that decreased Cx43 expression in blood vessels contributes significantly to the abnormal development of blood vessels in diabetic retinopathy [20].

## 3. The Interaction between Connexin and VEGF

### 3.1. The Effect of Connexin on VEGF

In angiogenesis, the VEGF family is the primary regulating factor, with VEGFA playing a significant role. VEGFA is used in conjunction with its receptors VEGFR1 and VEGFR2 to activate the passage, thereby promoting vascular endothelial cell survival, proliferation, migration, and ultimately the formation of new blood vessels [21]. Connexin has been shown to influence the release of VEGF from a variety of cell types.

Connexin43 hemichannels modulate inflammation in human retinal pigment epithelium cells by regulating ATP release. Under the condition of high glucose, the release of IL-6, IL-8, MCP-1, sICAM-1, and VEGF increased when human retinal pigment epithelial cells were stimulated with IL-1*β* and TNF-*α*, but the expression of these cytokines decreased significantly after using a Cx43 blocker peptide5. It has been proposed that blocking the function of Cx43 can inhibit inflammation [22]. However, under conditions of oxidative stress, overexpression of Cx43 in retinal pigment epithelial cells reduces VEGF gene and protein expression [23–25]. Diabetic model mice showed retinal angiogenesis and overexpression of vascular endothelial growth factor, which was related to the overexpression of Cx43 in the retina [26]. After coculture of outgrowth endothelial cells and osteoblasts, the researchers discovered that intercellular communication via Cx43 gap junction was improved, as was VEGF expression by osteoblasts. Thus they considered that gap junction intercellular communication plays an important role in cell development [27]. Cx43 also plays a role in the development of chronic cerebral perfusion injury by stimulating angiogenesis. The researchers discovered that, 30 days following bilateral carotid artery stenosis in mice, wild-type mice had more cerebral blood flow than Cx43+/− mice, whereas Cx43+/− mice had lower levels of angiogenesis-related proteins VEGF, HIF-1*α*, and its pathway protein p-AKT. These proteins are also differentially expressed in brain microvascular endothelial cells. They used siRNA to inhibit Cx43 expression in cells, and the expression of these proteins significantly decreased in these cells compared to untreated cells when stimulated by angiogenesis [28].

It is well established that vascular endothelial cells not only release VEGF but also absorb it from the external environment via an autocrine/paracrine pathway [29]. Certain cells generate VEGF and can drive vascular endothelial cell growth in a variety of ways, resulting in angiogenesis. Numerous investigations have discovered that interfering with intercellular communication can impair the ability of vascular endothelial cells to absorb VEGF. The most critical phase in angiogenesis is the migration of vascular endothelial cells. Monocytes have a role: they move to blood arteries and release a significant quantity of proinflammatory cytokines that stimulate vascular endothelial cells [30]. Researchers discovered that when an ischemic brain injury occurs, bone marrow monocytes can stimulate angiogenesis. When bone marrow monocytes and vascular endothelial cells are cocultured, the VEGF secreted by monocytes will be uptook by the cells around them. When inhibiting Cx43 function, this effect disappears [31]. According to several studies, glioblastoma promotes angiogenesis via the Cx43 gap junction. Cx43 gap junctions are required for the transport of VEGF from the glioblastoma to endothelial cells and subsequent tube formation in endothelial cells [32–34]. When vascular endothelium is injured, it can be repaired in two ways, by expanding and proliferating nearby endothelial cells or by transforming endothelial progenitor cells in the blood circulation. These paracrine vascular progenitor cells stimulate angiogenesis by releasing VEGF. However, studies have shown that, knocking down Cx43 expression in these cells, the release of VEGF by vascular progenitor cells was decreased, hence inhibiting angiogenesis [35].

However, in some cases, Cx43 has the reverse effect on VEGF expression, and decreasing Cx43 expression promotes VEGF expression. Decreased expression of Cx43, particularly in tumor cells, not only promotes tumor migration but also promotes the increase of VEGF in the tumor environment, resulting in angiogenesis [14]. Researchers knocked down Cx43 expression in human breast cancer cells, which resulted in increased expression of VEGF and aided cancer cell growth [36]. In addition to human tumor cells, connexin was found to negatively regulate VEGFA in animal tumor cells. Reduced Cx43 expression increases VEGFA expression in mouse tumor cells, thereby promoting tumor angiogenesis. *In vivo* experiments confirmed that the Cx43 overexpressing tumor cells were implanted into mice. In comparison to the control group, both tumor size and blood vessel density were decreased [37]. Thus, connexin plays a critical role in tumor cells, not only in restricting tumor growth but also in reducing the formation of pathological blood arteries in tumor cells. In tumor microenvironment, the inhibition of connexin function promotes tumor growth (Table 1).

### 3.2. The Effect of VEGF on Connexin

Connexins can modulate the expression of vascular endothelial growth factors, while VEGF also affects connexin function. Connexin expression on the surface of the majority of cells increased in response to high VEGF concentrations. Interfering with VEGF expression and function can have a detrimental effect on connexin. To begin, an increase in VEGF can enhance connexin expression. Gap junctions are significantly enhanced in U-251 glioblastoma multiforme cells, following VEGF treatment [48]. VEGF promotes the expression of Cx43 in endothelial progenitor cells. At the same time, silencing Cx43 activity inhibits VEGF's ability to promote endothelial progenitor cell homing and reendothelialization [39]. Increased expression of VEGF120 results in increased expression of Cx43, which decreases heart rate and hypervascularizes the sinoatrial node [49]. The expression of Cx43 induced by VEGF can alter over time in some cases [50]. One research found that when primary porcine pulmonary artery endothelial cells were exposed to VEGF, the expression of Cx43 GJs significantly decreased over the first hour. Cx43 expression was restored to normal levels following prolonged incubation [51].

**Figure 1:**
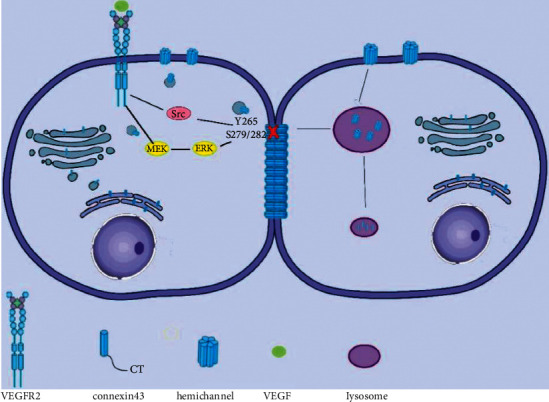
The connexin gene is transcribed and translated from the nucleus to the endoplasmic reticulum, where it undergoes simple modification. The connexin is then transfered to the Golgi apparatus, where it is assembled and finally transported to the cell membrane's surface. Every six connexins gather on the surface of the cell membrane to form a hemichannel. Additionally, hemichannels aid in signal communication between the cell and its surroundings. On the other hand, the two hemichannels in each cell are combined to form a gap junction, which may facilitate cell-cell communication. The CT terminal of the connexin plays an important role in its function, since it promotes endocytosis and aids in their entry into the lysosome for degradation. VEGF can regulate connexin function in two ways: It can stimulate the MEK/ERK pathway, hence inhibiting the phosphorylation of Cx43 protein at S279/282. The other is Src-mediated phosphorylation of Y265. Thus, by modifying the gap junction protein's CT terminus, its function can be modulated.

As previously stated, connexin's function is largely determined by its CT terminal; thus, it is hypothesized that VEGF affects connexin's quantity and function via the CT terminal. The researchers discovered that VEGF can phosphorylate the C-terminus of Cx43, impairing gap junction intercellular communication. At the same time, it can result in the internalization of Cx43 and accelerate its decomposition. Pretreatment of primary porcine pulmonary artery endothelial cells with VEGF can promote Cx43 C-terminal tail phosphorylation, leading to GJs internalization. This phenomenon is a result of VEGF activating a hierarchical kinase cascade that includes PKC and MAPK [51]. VEGF165 can also cause Cx43 phosphorylation in ovine uterine artery endothelial cells through activating Src family kinase signaling and MEK/ERK signaling, as a result of inhibiting pregnancy-adapted Ca^2+^ burst function [52]. VEGF induces the phosphorylation of connexin 43 in HUVECs, hence affecting gap junction intercellular communication [53]. VEGF has been shown to impair Cx43 gap junction intercellular communication and promote Cx43 endocytosis and phosphorylation in coronary capillary endothelium [54]. VEGF activates VEGFR2 in both Ea.hy926 and HUVECs and then induces Cx43 phosphorylation via Src family kinase signaling and the Erk family of MAP kinases. Therefore, the GJIC function of Cx43 is blocked. However, after approximately one hour, this function can be reversed. Researchers hypothesize that VEGF-induced GJC blockade may act by increasing endothelial permeability in injured blood vessels [55]. VEGF inhibits the Ca^2+^ burst in pregnant sheep by phosphorylating Cx43 at Tyr-265 and Ser-279/282 via the VEGFR2 Src and ERK pathways. Additionally, VEGF inhibits GJC function by activating VEGFR2 [56]. A variety of cell-cell junctions can be formed between endothelial cells or with other cells, and these junctions can be affected by various proangiogenesis growth factors. Some related studies have found that gap junction communication in vascular endothelial cells is disrupted during the early stages of VEGF stimulation and then resumes 1-2 hours after treatment. This disruption is attributed to VEGF's effect on the phosphorylation state of Cx43, which ultimately affects the permeability of vascular endothelial cells [55] (Figure 1 and Table 2).

## 4. The Interaction between Connexin and bFGF

FGFs can stimulate the growth of a wide variety of cell types. They can help preserve the function of vascular endothelial cells and encourage their proliferation during angiogenesis. FGF can effectively stimulate the proliferation of vascular endothelial cells and the formation of new blood vessels during the wound repair process [66, 67]. FGF is a potential angiogenic factor in cornea, and FGF can cause corneal neovascularization by combining with VEGF [68]. In corneal neovascularization, bFGF upregulates the activity of fibroblasts and causes tissue to release a large amount of VEGF and MMP14, which can induce corneal neovascularization [69, 70]. In a rat model of prolactinoma, carbenoxolone inhibited the Cx43 gap junction, hence decreasing bFGF expression [41]. Cx43 overexpression in MSCs promotes the release of VEGF and bFGF in the infarcted heart. However, when gap junction inhibitors or agonists are used, there is no difference between these two angiogenic factors [42]. bFGF has a variable effect on gap junctions and hemichannels in various cells. In C6 glioma cells, bFGF and LPS inhibit the function of the Cx43 gap junction but have a beneficial effect on the function of the hemichannels. However, bFGF and LPS inhibited the function of hemichannels in HeLa cells [57]. Angiogenic factors stimulate gap junction heterocellular communication between endothelial and hematologic malignant cells [58]. During wound healing, bFGF enhances wound healing in part by promoting angiogenesis. Cx43 and GJIC expression are increased because of the function of bFGF in skin fibroblasts [59]. The same phenomenon occurs in cardiac fibroblasts [61] (Tables 1 and 2).

## 5. The Interaction between Connexin and EGF

EGF cannot directly regulate the growth of blood vessels; rather, it is considered an indirect regulator of angiogenesis. Activation of the EGFR signaling pathway can increase the expression of VEGF, thus affecting angiogenesis [7, 71, 72]. Numerous studies have discovered that connexin and EGF interact to regulate cell proliferation. In ovarian cancer cells, Cx43 gene knockout promoted EGF-induced cell proliferation, but overexpression of Cx43 decreased EGF's influence on ovarian cancer cell proliferation [73]. Cx43 expression and the EGF/EGFR signaling pathway coregulate neural progenitor cell self-renewal and differentiation. Among these, EGF stimulates the expression of Cx43 in cells, whereas inhibition of Cx43 reduces the effect of EGF on cell proliferation [74]. Cx43 inhibits EGF and FGF signaling by reducing Src activity, impairing the proliferation and differentiation of neural progenitor cells [75]. EGF regulates mouse embryonic stem cell proliferation by promoting CX43 phosphorylation [76]. EGF inhibits gap junctional communication between cells by phosphorylating CX43 via MAPK [77]. As previously stated, activation of certain tyrosine kinase receptor proteins can result in phosphorylation of the CT terminal of gap junction proteins, impairing connexin function. Through the MAPK pathway, EGF alters the gating properties of Cx43, hence accelerating its internalization and degradation [13, 78].

## 6. The Interaction between Connexin and PDGF

PDGF plays the role of recruiting pericytes and vascular smooth muscle cells in the process of neovascularization and develops neovascularization to stable and mature vessels [79]. PDGF can modulate the function of vessels. It has two receptors, PDGF*α* and PDGF*β*, and can stimulate neovascularization by regulating VEGF release and the proliferation of cells around blood vessels. Its most important role is to establish stable functional blood vessels by angiogenesis, which recruits surrounding cells [80, 81]. As an important regulator of angiogenesis, it also interacts with connexin in the formation of new vessels. PDGF-BB can significantly alter blood perfusion and vascular reactivity in rats during hemorrhagic shock; however, this characteristic is dependent on the integrity of connexin in vascular endothelial and myoendothelial cells [82]. In the presence of VEGF and PDGF, nanofiber-expanded human cord blood-derived hematopoietic stem cells can induce the expression of Cx43 and angiogenic factors, thereby improving cardiac function following myocardial infarction [83]. PDGF can modulate the function of the Cx43 gap junction and accelerate its internalization and degradation via MAPK and PKC signaling pathways [78, 84].

## 7. The Interaction between Connexin and TNF-*α*

As a proinflammatory factor, TNF-*α* plays an important role in both corneal inflammation and retinal inflammation. The occurrence of inflammation will also cause neovascularization. TNF-*α*, in conjunction with VEGF, can promote the growth of blood vessels. TNF-*α* can destroy the sugar calyx layer of endothelial cells and enhance vascular permeability, resulting in surrounding tissue edema [85]. At the same time, TNF-*α* may recruit leukocytes to the inflammatory site via the release of chemokines, which in turn release a variety of growth factors and cytokines that stimulate angiogenesis [86]. Researchers have also discovered an interaction between Cx43 and TNF-*α*. In EA.hy 926 cells, Cx43 hemichannel function was improved following treatment with high glucose and IL-1*β*/TNF-*α*. Additionally, this impact can be abrogated by knocking down Cx43 expression [87]. TNF-*α* production is increased during preeclampsia, which inhibits Cx43 function via the Src pathway. This modification results in decreased vasodilation of the uterine arteries [64]. Both Cx43 and GJIC are decreased in human corneal fibroblasts via the ubiquitin-proteasome pathway in response to TNF-*α* stimulation [63]. Both the astrocytic Cx43 gap junctions and GJIC are downregulated during inflammation. TNF-*α* activates the ubiquitin-proteasome system, which regulates Cx43 and GJIC expression via a JNK-dependent pathway [88] (Table 2).

## 8. The Interaction between Connexin and MMP

One key step in corneal neovascularization is the hydrolysis of vascular endothelial basement membrane and surrounding extracellular matrix proteins, which is mainly accomplished by MMP [79]. The proteolytic function of matrix metalloproteinases has been shown to induce angiogenesis, with MMP9 and MMP2 being implicated in this process [89]. Cx43 regulates these two proteins. MMP2 is thought to be related to the apoptosis-promoting effect of capillaries in diabetic retinopathy. Under this condition, the activation of MMP2 reduces the expression of Cx43 in mitochondria [90, 91]. Ulinastatin affects monocyte-endothelial adhesion by blocking ROS transfer between HUVECs mediated by Cx43, resulting in decreased MMP2 and MMP9 expression [43]. Oleamide, a Cx43 gap junction inhibitor, has been shown to suppress MMP9 expression in MDA-MB-231 breast cancer cells [44]. Hypoxia increased MMP-9 activation and decreased Cx43 protein expression in rat H9C2 cardiomyocytes. Doxycycline, an MMP inhibitor, can alter this phenomenon [65] (Table 1 and Table 2).

## 9. Effect of Connexin on Vascular Permeability

Endothelial cells' barrier function is very important for the function and integrity of blood vessels. Angiogenesis satisfies the growth of the body's organs and tissues under physiological conditions by supplying sufficient nutrients to these parts. In pathological situations, vascular leakage results in tissue edema attracting a high number of inflammatory cells to the injured site and eventually results in extracellular matrix reconstruction and tissue damage [96, 97]. Adhesive connections and tight junctions maintain the endothelial barrier. VE-cadherin plays an important role in maintaining barrier stability. When VE-cadherin is destroyed in embryos, early embryonic death occurs, whereas adults experience vascular leakage and bleeding [96, 98]. Vascular permeability is also highly dependent on the function of tight junction proteins; when these proteins are affected, vascular permeability increases, resulting in tissue edema. The key tight junction proteins are ZO-1 and occludin [99]. The difference while the absence of tight junction protein does not affect embryonic development can disrupt the blood-brain barrier, leading to death from cerebral hemorrhage [100, 101]. The destruction of vascular barriers occurs in many neovascularization diseases. This is mostly because several growth factors, including VEGF, increase vascular permeability in these inflammatory diseases. Cx43 also has an effect on the expression of compact protein during angiogenesis. Cx43 expression decreases ZO-1 and occludin expression and increases vascular permeability, resulting in vascular leakage.

Cx43 and ZO-1 have been shown to interact on the cell membrane. After Cx43 is synthesized in the cell, it migrates to the cell membrane surface in the form of a connexin or hemichannel and interacts with cytoskeleton proteins and ZO-1. Phosphorylation of cytoskeleton proteins can contribute to the stability of hemichannels and gap junctions [102–105]. In retinal diseases, destroying blood-retinal barrier stability has a great impact on the retinal structure. This stability depends on the integrity of tight junctions between cells [106, 107]. A significant pathogenic aspect of diabetic retinopathy is the destruction of the blood-retinal barrier, which is associated with decreased expression of tight junction protein. It has been discovered that when glucose levels are elevated, Cx43 expression in retinal endothelial cells decreases, and the expression of ZO-1 and occludin protein decreases, resulting in vascular leakage and fundus hemorrhage [108]. In addition, researchers also found that changes in the expression of connexin in mitochondria also affect vascular function. Connexin in mitochondria has been proved to regulate apoptosis. Retinal endothelial cells in high glucose environment expressing mitochondrial damaging and decreasing of mitochondrial connexin [90, 109, 110]. According to a previous study, Cx43 can affect the rate of wound healing in cerebral vascular endothelial cells by forming a complex with ZO-1 and altering the dynamics of the cytoskeleton [111]. Cx43's carboxyl tail interacts with ZO-1's PDZ-2 domain [111–113]. In corneal and skin injury models, disrupting the binding of Cx43 and ZO-1 can promote epithelial repair [114–116]. Although the blood-brain barrier plays an important role in the central nervous system, when inflammatory factors invade, the permeability of microvascular endothelial cells in the blood-brain barrier increases. Researchers have found that when experimental mice were stimulated with LPS, the permeability of the blood-brain barrier increased and the detection of associated proteins, occludin-1 and Cx43, significantly decreased [117]. Increased vascular permeability is a significant consequence of sepsis, and some researchers have discovered that changes in Cx43 are associated with increased vascular permeability. Increased expression of Cx43 was observed in both vascular endothelial cells and tissues in the rat sepsis model and LPS-induced increase in vascular permeability in rat pulmonary vein endothelial cells, and the mechanism was related to the Rock1-MLC20 phosphorylation pathway [118]. Cx43 upregulates the expression of osteopontin to increase vascular permeability by downregulating tight junction proteins ZO-1 and Claudin-5 [45]. Cx43 has been shown to stabilize blood vessels in the brain in the presence of chronic cerebral hypoperfusion. Mice were subjected to bilateral carotid artery stenosis. Using a microscopic angiography method to observe cerebral cortical veins, it was discovered that vascular leakage increased and tight junction related proteins decreased in Cx43+/− mice. ZO−1 and Claudin-5 expressions were much lower in Cx43+/−mice than in wild mice. There was also a difference in the expression of ZO−1 and Claudin-5 in cerebral microvessels of Cx43+/− mice. siRNA was used to inhibit Cx43 expression, and the expression of tight junction-related proteins significantly decreased between treated and untreated cells in response to angiogenesis stimulation [28]. Cx43 is involved in familial type 3 cerebral cavernous hemangioma by influencing tight junction protein expression and thereby improving the permeability of the brain endothelial cell barrier. In the brain tissue of mice with this disease, Cx43 and GJIC were increased, angiogenesis occurred, and vascular leakage also increased. The researchers discovered that Cx43 expression increased in cerebral vascular endothelial cells, but the expressions of tight junction proteins, Claudin-5 and ZO-1, decreased. This demonstrates that increased Cx43 expression promotes vascular growth and causes vascular leakage in cerebral hemangiomatosis [46]. This is in contrast to the observation that Cx43 maintains the vascular structure and enhances tight junction protein expression in chronic cerebrovascular injury diseases. We hypothesize that the blood vessels formed by hemangiomas are neovascularization, the structure of these vessels is unstable, and Cx43 inhibits the expression of a compact protein. Additionally, in chronic vascular disease, when blood vessels tend to be stable, Cx43 plays a role in vessel stabilization. In the early stage of LPS-induced lung injury, the expression of Cx43 in microvascular endothelial cells and vascular leakage increased. In the early stage, the expression of tight junction-associated protein VE-cadherin decreased and showed a negative correlation with the change in Cx43. Therefore, Cx43 promotes vascular leakage by inhibiting the VE-cadherin expression during the early stages of inflammation [47]. A related study found that ZO-1 can also regulate the rate, size, and distribution of Cx43 formation [119] (Table 1).

## 10. Connexin Affects Angiogenesis by Affecting the Transmission of miRNA between Cells

miRNA is a small noncoding RNA, derived from introns or exons. Numerous studies have established that miRNA plays an important role in vascular-related diseases. In angiogenesis-related diseases, miRNA expression is aberrant, which plays an important role in the regulation of vascular endothelial cells, periendothelial cells, and angiogenic signals. Therefore, by altering the expression level of miRNA, researchers can treat ischemic diseases and inhibit tumor growth via the angiogenic function and antivascular functions of miRNA, respectively [120, 121]. Many miRNAs play an important role in the process of corneal neovascularization. It was found that miRNA-31, miRNA-122, miRNA-126, miRNA-132, miRNA-133b, miRNA-145, miRNA-155, miRNA-184, and miRNA-205 were related to corneal neovascularization. Moreover, the miRNA related to neovascularization was detected by gene chip technology in the cornea of inflammatory rats. Researchers found that miRNA-21, miRNA-27a, miRNA-29, miRNA-142, and miRNA-1224 were significantly changed in the cornea of inflammatory rats [122]. Cx43, as an intercellular signaling protein, can influence miRNA expression and, in some cases, its transmission between cells.

miR-200b, a negative regulator, is conveyed to HUVECs via the Cx43 gap junction in rat bone marrow mesenchymal stem cells. Additionally, decreased miR-200b expression in BMSCs reduces the expression of VEGFA, which is involved in osteogenic differentiation. Increased miR-200b expression has been shown to inhibit vascular endothelial cells' angiogenic capacity [93]. In malignant glioblastomas, miR-5096, a proangiogenesis microRNA, can be transported from malignant glioblastomas to endothelial cells via the Cx43 gap junction. Increasing expression of the Cx43 protein can facilitate the transfection of this microRNA from glioblastoma to endothelial cells, resulting in an aggressive growth of the blood vessels [92]. Similarly, miR-145 and miR-5096 were also discovered to inhibit angiogenesis and tumor growth when transmitted between human microvascular endothelial cells and glioblastomas via the Cx43 gap junction [94]. When colon cancer cells are cocultured with human microvascular endothelial cells, functional Cx43 gap junctions between the two cells are formed. miR-145 transfered from colon cancer cells to human microvascular endothelial cells via this gap junction, where it suppresses angiogenesis [95] (Table 3).

## 11. Conclusions

Numerous studies have demonstrated that altering connexin in vascular endothelial cells affects their function, hence impacting neovascularization. However, the mechanism through which the number and function of connexin affect angiogenesis in vascular endothelial cells is unknown. Variations in the expressions of angiogenic factors are implicated in both physiological and pathological angiogenesis. As an important visual organ, the eye is seriously affected by pathological neovascularization in many cases. At present, the antineovascularization drugs used in ophthalmology are limited, so we need to seek more methods to solve this issue. Therefore, in this article, we summarize the interaction between connexin and angiogenic factors. Among them, VEGF is the most important growth factor, and it has been discovered that connexin can regulate its expression. Connexin primarily influences vascular growth in an inflammatory environment by inhibiting the release of VEGF from other cells surrounding vascular endothelial cells. On the other hand, VEGF alters the function of connexin or promotes its degradation mostly through its CT terminus. Other angiogenic factors similarly interact with connexin. As is well known, vascular leakage is another significant aspect of pathogenic neovascularization. Numerous studies have established a strong correlation between the CT end of gap junction protein and the tight junction protein ZO-1. Connexin exerts this effect on ZO-1 expression, hence regulating vascular leakage. Therefore, we need to conduct additional research on connexin's role in angiogenesis, particularly how it affects the expression of angiogenic factors. We hypothesize that altering the function of gap junctions may provide a mechanism for regulating angiogenesis.

## Figures and Tables

**Table 1:** Effect of Cx43 on angiogenic factors.

Connexin type	Angiogenic factors	Comment
Cx43	VEGF [32]	Cx43 facilitated the transport of VEGF from glioblastoma to endothelial cells
Cx43 GJ	VEGF [31]	BM-MNC promoted VEGF uptake into HUVEC through gap junction-mediated pathway
CX43 HC	VEGF [22]	Inhibition of Cx43 prevented the release of VEGF from human adult retinal pigment epithelial cells
Cx43 HC and GJ	VEGF [38]	Inhibition of Cx43 upregulated VEGF mRNA expression
Cx43 GJ	VEGF [39]	Inhibition of Cx43 blocked the effect of VEGF on endothelial progenitor cells homing and reendothelialization
Cx43	VEGF [24, 25]	Suppression of Cx43 reduced VEGF secretion from human endometrial stromal cells
Cx43	VEGF [36]	Suppression of Cx43 expression in human breast cancer cells increased VEGF expression
Cx43	VEGF [40]	Inhibition of Cx43 gap junction function upregulated mRNA and protein expression of VEGF in gingival fibroblasts
Cx43	VEGF [23]	During oxidative stress conditions, overexpression of Cx43 in retinal pigment epithelial cells reduced gene and protein expression of VEGF
Cx43	VEGF [35]	Downregulation of Cx43 alleviated angiogenesis and VEGF secretion from endothelial progenitor cells
Cx43	VEGF [37]	Downregulation of Cx43 in tumor cells promoted VEGF expression leading to angiogenesis
Cx43	VEGF [28]	Suppression of Cx43 expression in mouse brain microvascular endothelial cells reduced the expression of VEGF
Cx43	bFGF [41]	Downregulation of Cx43 expression decreased bFGF expression in prolactinoma cells
Cx43	bFGF and VEGF [42]	Overexpression of Cx43 in MSCs promoted the release of VEGF and bFGF from infarcted heart
Cx43	MMP2 and MMP9 [43]	Inhibition of the function of Cx43 in HUVECs downregulated MMP2 and MMP9 expression
Cx43	MMP9 [44]	Inhibition of Cx43 reduced MMP9 expression in MDA-MB-231 breast cancer cells
Cx43	Claudin-5 and ZO-1 [45]	Downregulation of Cx43 decreased the expression of Claudin-5 and ZO-1, thus increasing vascular permeability
Cx43	Claudin-5 and ZO-1 [28]	Cx43 stabilized blood vessels during chronic cerebral hypoperfusion. The expression of ZO-1 and Claudin-5 in Cx43+/− mice was downregulated
Cx43	Claudin-5 and ZO-1 [46]	Overexpression of Cx43 promoted vascular growth and vascular leakage
Cx43	VE-cadherin [47]	Downregulating Cx43 increased vascular leakage by decreasing expression of VE-cadherin

**Table 2:** Effect of angiogenic factors on Cx43.

Angiogenic factors	Connexin type	Comment
VEGF [52]	Cx43 GJIC	Activation of VEGFR2 by VEGF-E alone inhibited GJIC
VEGF [48]	Cx43 GJIC	EGF promoted GJIC in glioblastoma multiforme cells
VEGF [48]	Cx43 GJ	VEGF increased the expression of gap junction proteins in U-251 glioblastoma multiforme cells
VEGF [39]	Cx43	VEGF enhanced the expression of Cx43 in endothelial progenitor cells
VEGF [51]	Cx43 GJ	Exposure of primary porcine pulmonary artery endothelial cells to VEGF markedly suppressed Cx43 GJs within 1 hour
VEGF [52]	Cx43 GJ	VEGF induced Cx43 phosphorylation leading to a decline in pregnancy-adapted Ca^2+^ burst function
VEGF [49]	Cx43	VEGF120 increased expression of Cx43 in heart
VEGF [50]	Cx43 GJIC	VEGF improved intercellular communication at the Cx43 gap junction
VEGF [54]	Cx43 GJIC	In coronary capillary endothelium, VEGF disrupted intercellular communication and promoted Cx43 endocytosis and phosphorylation at the Cx43 gap junction
VEGF [53]	Cx43 GJIC	VEGF induced phosphorylation of connexin 43 to regulate gap junction intercellular communication in HUVECs
VEGF [55]	Cx43 GJIC	VEGF reversibly inhibited GJIC function in EA.hy926 cells and HUVECs
bFGF and LPS [57]	Cx43 GJ and HC	Inhibited Cx43 gap junctions but stimulated hemichannels in C6 glioma cells
bFGF and LPS [57]	Cx43 GJ and HC	Inhibited Cx43 hemichannels function in HeLa cells.
bFGF and VEGF [58]	Cx43 GJIC	Acted as angiogenic factors to increase gap junction heterocellular communication between endothelial cells and hematologic malignant cells
bFGF [59]	Cx43 expression and GJIC	bFGF stimulated GJIC and promoted Cx43 expression in skin fibroblasts
bFGF [60]	Cx43 expression and GJIC	bFGF stimulation increased Cx43 expression and GJIC in cortical progenitor cells
bFGF [61]	Cx43 expression and GJIC	bFGF stimulation increased Cx43 expression and GJIC in cardiac fibroblasts
TNF-*α* [62]	Cx43 HC	Under high glucose conditions, TNF-*α* increased Cx43 hemichannel function in EA.hy926 cells
TNF-*α* [63]	Cx43 GJ and GJIC	TNF-*α* decreased the amount of Cx43 gap junction protein and GJIC in corneal fibroblasts
TNF-*α* [64]	Cx43 GJIC	TNF-*α* decreased Cx43 GJIC in ovine uterine artery endothelial cells and HUVECs
MMP9 [65]	Cx43	The hypoxia-induced downregulation of Cx43 protein was markedly attenuated by doxycycline, an inhibitor of MMP

**Table 3:** Effect of Cx43 on microRNA.

microRNA	Connexin type	Comment
miR-5096 [92]	Cx43 GJ	It is transferred from glioblastoma to endothelial cell to regulate angiogenesis
miR-200b [93]	Cx43 GJ	It is transferred from rat bone-marrow derived mesenchymal stem cells to HUVECs to regulate osteogenesis and angiogenesis
miR-145 [94]	Cx43 GJ	It is transferred from glioblastoma to HMECs to regulate angiogenesis
miR-145 [95]	Cx43 GJ	It is transferred from colon cancer cells to HMECs to regulate angiogenesis

## References

[1] Carmeliet P., Jain R. K. (2011). Molecular mechanisms and clinical applications of angiogenesis. *Nature*.

[2] Hadrian K., Willenborg S., Bock F., Cursiefen C., Eming S. A., Hos D. (2021). Macrophage-mediated tissue vascularization: similarities and differences between cornea and skin. *Frontiers in Immunology*.

[3] Zhu L., Lama S., Tu L., Dusting G. J., Wang J.-H., Liu G.-S. (2021). TAK1 signaling is a potential therapeutic target for pathological angiogenesis. *Angiogenesis*.

[4] Risau W. (1997). Mechanisms of angiogenesis. *Nature*.

[5] Mentzer S. J., Konerding M. A. (2014). Intussusceptive angiogenesis: expansion and remodeling of microvascular networks. *Angiogenesis*.

[6] Rohlenova K., Goveia J., García-Caballero M. (2020). Single-cell RNA sequencing maps endothelial metabolic plasticity in pathological angiogenesis. *Cell Metabolism*.

[7] Cook K. M., Figg W. D. (2010). Angiogenesis inhibitors: current strategies and future prospects. *CA: A Cancer Journal for Clinicians*.

[8] Söhl G., Willecke K. (2004). Gap junctions and the connexin protein family. *Cardiovascular Research*.

[9] Goodenough D. A., Paul D. L. (2003). Beyond the gap: functions of unpaired connexon channels. *Nature Reviews Molecular Cell Biology*.

[10] Alexander D., Goldberg G. (2003). Transfer of biologically important molecules between cells through gap junction channels. *Current Medicinal Chemistry*.

[11] Pohl U. (2020). Connexins: key players in the control of vascular plasticity and function. *Physiological Reviews*.

[12] Pogoda K., Kameritsch P., Mannell H., Pohl U. (2019). Connexins in the control of vasomotor function. *Acta Physiologica*.

[13] Leithe E., Mesnil M., Aasen T. (2018). The connexin 43 C-terminus: a tail of many tales. *Biochimica et Biophysica Acta (BBA)—Biomembranes*.

[14] Okamoto T., Usuda H., Tanaka T., Wada K., Shimaoka M. (2019). The functional implications of endothelial gap junctions and cellular mechanics in vascular angiogenesis. *Cancers*.

[15] Okamoto T., Suzuki K. (2017). The role of gap junction-mediated endothelial cell-cell interaction in the crosstalk between inflammation and blood coagulation. *International Journal of Molecular Sciences*.

[16] Walker D. L., Vacha S. J., Kirby M. L., Lo C. W. (2005). Connexin43 deficiency causes dysregulation of coronary vasculogenesis. *Developmental Biology*.

[17] Rhee D. Y., Zhao X.-Q., Francis R. J. B., Huang G. Y., Mably J. D., Lo C. W. (2009). Connexin 43 regulates epicardial cell polarity and migration in coronary vascular development. *Development*.

[18] Clauss S. B., Walker D. L., Kirby M. L., Schimel D., Lo C. W. (2006). Patterning of coronary arteries in wildtype and connexin43 knockout mice. *Developmental Dynamics: An Official Publication of the American Association of Anatomists*.

[19] Teixeira T. F., da Silva T. C., Fukumasu H., de Lima C. E., Lúcia Zaidan Dagli M., Guerra J. L. (2007). Histological alterations in the livers of Cx43-deficient mice submitted to a cholestasis model. *Life Sciences*.

[20] Bobbie M. W., Roy S., Trudeau K. (2010). Reduced connexin 43 expression and its effect on the development of vascular lesions in retinas of diabetic mice. *Investigative Opthalmology & Visual Science*.

[21] Le T., Kwon S.-M. (2021). Vascular endothelial growth factor biology and its potential as a therapeutic target in rheumatic diseases. *International Journal of Molecular Sciences*.

[22] Mugisho O. O., Green C. R., Kho D. T. (2018). The inflammasome pathway is amplified and perpetuated in an autocrine manner through connexin43 hemichannel mediated ATP release. *Biochimica et Biophysica Acta (BBA)—General Subjects*.

[23] Pocrnich C. E., Shao Q., Liu H. (2012). The effect of connexin43 on the level of vascular endothelial growth factor in human retinal pigment epithelial cells. *Graefe’s Archive for Clinical and Experimental Ophthalmology*.

[24] Yu J., Wu J., Bagchi I. C., Bagchi M. K., Sidell N., Taylor R. N. (2011). Disruption of gap junctions reduces biomarkers of decidualization and angiogenesis and increases inflammatory mediators in human endometrial stromal cell cultures. *Molecular and Cellular Endocrinology*.

[25] Laws M. J., Taylor R. N., Sidell N. (2008). Gap junction communication between uterine stromal cells plays a critical role in pregnancy-associated neovascularization and embryo survival. *Development*.

[26] Mugisho O. O., Green C. R., Zhang J. (2017). Immunohistochemical characterization of Connexin43 expression in a mouse model of diabetic retinopathy and in human donor retinas. *International Journal of Molecular Sciences*.

[27] Herzog D. P., Dohle E., Bischoff I., Kirkpatrick C. J. (2014). Cell communication in a coculture system consisting of outgrowth endothelial cells and primary osteoblasts. *BioMed Research International*.

[28] Yu W., Jin H., Sun W. (2021). Connexin43 promotes angiogenesis through activating the HIF-1alpha/VEGF signaling pathway under chronic cerebral hypoperfusion. *Journal of Cerebral Blood Flow and Metabolism*.

[29] Lee S., Chen T. T., Barber C. L. (2007). Autocrine VEGF signaling is required for vascular homeostasis. *Cell*.

[30] Nowak-Sliwinska P., Alitalo K., Allen E. (2018). Consensus guidelines for the use and interpretation of angiogenesis assays. *Angiogenesis*.

[31] Kikuchi-Taura A., Okinaka Y., Takeuchi Y. (2020). Bone marrow mononuclear cells activate angiogenesis via gap junction-mediated cell-cell interaction. *Stroke*.

[32] Peleli M., Moustakas A., Papapetropoulos A. (2020). Endothelial-tumor cell interaction in brain and CNS malignancies. *International Journal of Molecular Sciences*.

[33] Zhang W., DeMattia J. A., Song H., Couldwell W. T. (2003). Communication between malignant glioma cells and vascular endothelial cells through gap junctions. *Journal of Neurosurgery*.

[34] Thuringer D., Boucher J., Jego G. (2016). Transfer of functional microRNAs between glioblastoma and microvascular endothelial cells through gap junctions. *Oncotarget*.

[35] Wang H.-H., Su C.-H., Wu Y.-J. (2013). Reduction of connexin43 in human endothelial progenitor cells impairs the angiogenic potential. *Angiogenesis*.

[36] Shao Q., Wang H., McLachlan E., Veitch G. I., Laird D. W. (2005). Down-regulation of Cx43 by retroviral delivery of small interfering RNA promotes an aggressive breast cancer cell phenotype. *Cancer Research*.

[37] Wang W.-K., Chen M.-C., Leong H.-F., Kuo Y.-L., Kuo C.-Y., Lee C.-H. (2014). Connexin 43 suppresses tumor angiogenesis by down-regulation of vascular endothelial growth factor via hypoxic-induced factor-1*α*. *International Journal of Molecular Sciences*.

[38] Tarzemany R., Jiang G., Jiang J. X., Larjava H., Häkkinen L. (2017). Connexin 43 hemichannels regulate the expression of wound healing-associated genes in human gingival fibroblasts. *Scientific Reports*.

[39] Li L., Liu H., Xu C. (2017). VEGF promotes endothelial progenitor cell differentiation and vascular repair through connexin 43. *Stem Cell Research & Therapy*.

[40] Roy S., Jiang J. X., Li A.-F., Kim D. (2017). Connexin channel and its role in diabetic retinopathy. *Progress in Retinal and Eye Research*.

[41] Wang H., Gao B., Zhang Y., Xu H. (2016). Effects of inhibiting connexin43 on expression of fibroblast growth factor in prolactinomas in rats. *Neurological Research*.

[42] Wang D.-G., Zhang F.-X., Chen M.-L., Zhu H.-J., Yang B., Cao K.-J. (2014). Cx43 in mesenchymal stem cells promotes angiogenesis of the infarcted heart independent of gap junctions. *Molecular Medicine Reports*.

[43] Ding H., Jiang Y., Jiang Y., Yuan D., Xiao L. (2020). Ulinastatin attenuates monocyte-endothelial adhesion via inhibiting ROS transfer between the neighboring vascular endothelial cells mediated by Cx43. *American Journal of Tourism Research*.

[44] Zibara K., Awada Z., Dib L. (2015). Anti-angiogenesis therapy and gap junction inhibition reduce MDA-MB-231 breast cancer cell invasion and metastasis in vitro and in vivo. *Scientific Reports*.

[45] Zhang J., Yang G., Zhu Y., Peng X., Li T., Liu L. (2018). Relationship of Cx43 regulation of vascular permeability to osteopontin-tight junction protein pathway after sepsis in rats. *American Journal of Physiology—Regulatory, Integrative and Comparative Physiology*.

[46] Johnson A. M., Roach J. P., Hu A. (2018). Connexin 43 gap junctions contribute to brain endothelial barrier hyperpermeability in familial cerebral cavernous malformations type III by modulating tight junction structure. *FASEB Journal*.

[47] Kandasamy K., Escue R., Manna J., Adebiyi A., Parthasarathi K. (2015). Changes in endothelial connexin 43 expression inversely correlate with microvessel permeability and VE-cadherin expression in endotoxin-challenged lungs. *American Journal of Physiology—Lung Cellular and Molecular Physiology*.

[48] Theiss C., Krcek R., Latzer P., Adamietz I., Bühler H. (2017). Influence of vascular endothelial growth factor and radiation on gap junctional intercellular communication in glioblastoma multiforme cell lines. *Neural Regeneration Research*.

[49] Calkoen E. E., Vicente-Steijn R., Hahurij N. D. (2015). Abnormal sinoatrial node development resulting from disturbed vascular endothelial growth factor signaling. *International Journal of Cardiology*.

[50] Wuestefeld R., Chen J., Meller K., Brand-Saberi B., Theiss C. (2012). Impact of vegf on astrocytes: analysis of gap junctional intercellular communication, proliferation, and motility. *Glia*.

[51] Nimlamool W., Andrews R. M. K., Falk M. M. (2015). Connexin43 phosphorylation by PKC and MAPK signals VEGF-mediated gap junction internalization. *Molecular Biology of the Cell*.

[52] Boeldt D. S., Grummer M. A., Yi F., Magness R. R., Bird I. M. (2015). Phosphorylation of Ser-279/282 and Tyr-265 positions on Cx43 as possible mediators of VEGF-165 inhibition of pregnancy-adapted Ca^2+^ burst function in ovine uterine artery endothelial cells. *Molecular and Cellular Endocrinology*.

[53] Zhang Y. W., Yao X. S., Murota S., Morita I. (2002). Inhibitory effects of eicosapentaenoic acid (EPA) on the hypoxia/reoxygenation-induced tyrosine kinase activation in cultured human umbilical vein endothelial cells. *Prostaglandins, Leukotrienes and Essential Fatty Acids*.

[54] Thuringer D. (2004). The vascular endothelial growth factor-induced disruption of gap junctions is relayed by an autocrine communication via ATP release in coronary capillary endothelium. *Annals of the New York Academy of Sciences*.

[55] Suarez S., Ballmer-Hofer K. (2001). VEGF transiently disrupts gap junctional communication in endothelial cells. *Journal of Cell Science*.

[56] Clemente L., Boeldt D. S., Grummer M. A. (2020). Adenoviral transduction of EGFR into pregnancy-adapted uterine artery endothelial cells remaps growth factor induction of endothelial dysfunction. *Molecular and Cellular Endocrinology*.

[57] De Vuyst E., Decrock E., De Bock M. (2007). Connexin hemichannels and gap junction channels are differentially influenced by lipopolysaccharide and basic fibroblast growth factor. *Molecular Biology of the Cell*.

[58] El-Sabban M. E., Merhi R. A., Haidar H. A. (2002). Human T-cell lymphotropic virus type 1–transformed cells induce angiogenesis and establish functional gap junctions with endothelial cells. *Blood*.

[59] Abdullah K. M., Luthra G., Bilski J. J. (1999). Cell-to-cell communication and expression of gap junctional proteins in human diabetic and nondiabetic skin fibroblasts: effects of basic fibroblast growth factor. *Endocrine*.

[60] Nadarajah B., Makarenkova H., Becker D. L., Evans W. H., Parnavelas J. G. (1998). Basic FGF increases communication between cells of the neocortex. *Journal of Neuroscience*.

[61] Doble B. W., Kardami E. (1995). Basic fibroblast growth factor stimulates connexin-43 expression and intercellular communication of cardiac fibroblasts. *Molecular and Cellular Biochemistry*.

[62] Jin L., Zhang Y., Liang W. (2020). Zeb1 promotes corneal neovascularization by regulation of vascular endothelial cell proliferation. *Communications Biology*.

[63] Kimura K., Nishida T. (2010). Role of the ubiquitin-proteasome pathway in downregulation of the gap-junction protein connexin43 by TNF-*α* in human corneal fibroblasts. *Investigative Opthalmology & Visual Science*.

[64] Ampey A. C., Boeldt D. S., Clemente L. (2019). TNF-alpha inhibits pregnancy-adapted Ca^2+^ signaling in uterine artery endothelial cells. *Molecular and Cellular Endocrinology*.

[65] Wu X., Huang W., Luo G., Alain L. A. (2013). Hypoxia induces connexin 43 dysregulation by modulating matrix metalloproteinases via MAPK signaling. *Molecular and Cellular Biochemistry*.

[66] Prudovsky I. (2021). Cellular mechanisms of FGF-stimulated tissue repair. *Cells*.

[67] Maciag T., Hoover G. A., Stemerman M. B., Weinstein R. (1981). Serial propagation of human endothelial cells in vitro. *Journal of Cell Biology*.

[68] Hajrasouliha A. R., Sadrai Z., Chauhan S. K., Dana R. (2012). b-FGF induces corneal blood and lymphatic vessel growth in a spatially distinct pattern. *Cornea*.

[69] Onguchi T., Han K. Y., Chang J.-H., Azar D. T. (2009). Membrane type-1 matrix metalloproteinase potentiates basic fibroblast growth factor-induced corneal neovascularization. *American Journal Of Pathology*.

[70] Pouw A. E., Greiner M. A., Coussa R. G. (2021). Cell-matrix interactions in the eye: from cornea to choroid. *Cells*.

[71] van Cruijsen H., Giaccone G., Hoekman K. (2005). Epidermal growth factor receptor and angiogenesis: opportunities for combined anticancer strategies. *International Journal of Cancer*.

[72] De Luca A., Carotenuto A., Rachiglio A. (2008). The role of the EGFR signaling in tumor microenvironment. *Journal of Cellular Physiology*.

[73] Qiu X., Cheng J.-C., Klausen C., Chang H.-M., Fan Q., Leung P. C. K. (2016). EGF-induced Connexin43 negatively regulates cell proliferation in human ovarian cancer. *Journal of Cellular Physiology*.

[74] Lemcke H., Kuznetsov S. A. (2013). Involvement of connexin43 in the EGF/EGFR signalling during self-renewal and differentiation of neural progenitor cells. *Cellular Signalling*.

[75] Talaverón R., Matarredona E., Herrera A., Medina J., Tabernero A. (2020). Connexin43 region 266-283, via Src inhibition, reduces neural progenitor cell proliferation promoted by EGF and FGF-2 and increases. *Astrocytic Differentiation*.

[76] Park J. H., Lee M. Y., Heo J. S., Han H. J. (2008). A potential role of connexin 43 in epidermal growth factor-induced proliferation of mouse embryonic stem cells: involvement of Ca^2+^/PKC, p44/42 and p38 MAPKs pathways. *Cell Proliferation*.

[77] Warn-Cramer B. J., Cottrell G. T., Burt J. M., Lau A. F. (1998). Regulation of connexin-43 gap junctional intercellular communication by mitogen-activated protein kinase. *Journal of Biological Chemistry*.

[78] Kanemitsu M. Y., Lau A. F. (1993). Epidermal growth factor stimulates the disruption of gap junctional communication and connexin43 phosphorylation independent of 12-0-tetradecanoylphorbol 13-acetate-sensitive protein kinase C: the possible involvement of mitogen-activated protein kinase. *Molecular Biology of the Cell*.

[79] Nicholas M. P., Mysore N. (2021). Corneal neovascularization. *Experimental Eye Research*.

[80] Laschke M. W., Elitzsch A., Vollmar B., Vajkoczy P., Menger M. D. (2006). Combined inhibition of vascular endothelial growth factor (VEGF), fibroblast growth factor and platelet-derived growth factor, but not inhibition of VEGF alone, effectively suppresses angiogenesis and vessel maturation in endometriotic lesions. *Human Reproduction*.

[81] Zhao Y., Adjei A. A. (2015). Targeting angiogenesis in cancer therapy: moving beyond vascular endothelial growth factor. *The Oncologist*.

[82] Liu L., Zhang J., Zhu Y. (2014). Beneficial effects of platelet-derived growth factor on hemorrhagic shock in rats and the underlying mechanisms. *American Journal of Physiology - Heart and Circulatory Physiology*.

[83] Das H., George J. C., Joseph M. (2009). Stem cell therapy with overexpressed VEGF and PDGF genes improves cardiac function in a rat infarct model. *PLoS One*.

[84] Hossain M. Z., Ao P., Boynton A. L. (1998). Platelet-derived growth factor-induced disruption of gap junctional communication and phosphorylation of connexin43 involves protein kinase C and mitogen-activated protein kinase. *Journal of Cellular Physiology*.

[85] Mark K. S., Trickler W. J., Miller D. W. (2001). Tumor necrosis factor-alpha induces cyclooxygenase-2 expression and prostaglandin release in brain microvessel endothelial cells. *Journal of Pharmacology and Experimental Therapeutics*.

[86] Sunderkötter C., Steinbrink K., Goebeler M., Bhardwaj R., Sorg C. (1994). Macrophages and angiogenesis. *Journal of Leukocyte Biology*.

[87] Sáez J. C., Contreras-Duarte S., Gómez G. I. (2018). Connexin 43 hemichannel activity promoted by pro-inflammatory cytokines and high glucose alters endothelial cell function. *Frontiers in Immunology*.

[88] Zhang F. F., Morioka N., Kitamura T., Hisaoka-Nakashima K., Nakata Y. (2015). Proinflammatory cytokines downregulate connexin 43-gap junctions via the ubiquitin-proteasome system in rat spinal astrocytes. *Biochemical and Biophysical Research Communications*.

[89] Fields G. B. (2019). Mechanisms of action of novel drugs targeting angiogenesis-promoting matrix metalloproteinases. *Frontiers in Immunology*.

[90] Mohammad G., Kowluru R. A., methods a. j. o. t. (2010). Matrix metalloproteinase-2 in the development of diabetic retinopathy and mitochondrial dysfunction. *Laboratory Investigation*.

[91] Mohammad G., Kowluru R. A., science v. (2011). Novel role of mitochondrial matrix metalloproteinase-2 in the development of diabetic retinopathy. *Investigative Opthalmology & Visual Science*.

[92] Buruiana A., Florian S. I., Florian A. I. (2020). The roles of miRNA in glioblastoma tumor cell communication: diplomatic and aggressive negotiations. *International Journal of Molecular Sciences*.

[93] Fan X., Teng Y., Ye Z., Zhou Y., Tan W. S. (2018). The effect of gap junction-mediated transfer of miR-200b on osteogenesis and angiogenesis in a co-culture of MSCs and HUVECs. *Journal of Cell Science*.

[94] Thuringer D., Solary E., Garrido C. (2017). The microvascular gap junction channel: a route to deliver MicroRNAs for neurological disease treatment. *Frontiers in Molecular Neuroscience*.

[95] Zefferino R., Piccoli C., Gioia S. D., Capitanio N., Conese M. (2019). Gap junction intercellular communication in the carcinogenesis hallmarks: is this a phenomenon or epiphenomenon?. *Cells*.

[96] Park-Windhol C., D’Amore P. A. (2016). Disorders of vascular permeability. *Annual Review of Pathology: Mechanisms of Disease*.

[97] Folkman J. (1995). Seminars in medicine of the beth Israel hospital, boston. Clinical applications of research on angiogenesis. *New England Journal of Medicine*.

[98] Smith R. O., Ninchoji T., Gordon E. (2020). Vascular permeability in retinopathy is regulated by VEGFR2 Y949 signaling to VE-cadherin. *Elife*.

[99] Harhaj N. S., Antonetti D. A. (2004). Regulation of tight junctions and loss of barrier function in pathophysiology. *International Journal of Biochemistry & Cell Biology*.

[100] Grund S., Grummer R. (2018). Direct cell (-) cell interactions in the endometrium and in endometrial pathophysiology. *International Journal of Molecular Sciences*.

[101] Tsukita S., Furuse M., Itoh M. (2001). Multifunctional strands in tight junctions. *Nature Reviews Molecular Cell Biology*.

[102] Solan J. L., Lampe P. D. (2020). Src regulation of Cx43 phosphorylation and gap junction turnover. *Biomolecules*.

[103] Márquez-Rosado L., Singh D., Rincón-Arano H., Solan J., Lampe P. J. (2012). CASK (LIN2) interacts with Cx43 in wounded skin and their coexpression affects cell migration. *Journal of Cell Science*.

[104] Rhett J. M., Jourdan J., Gourdie R. G. (2011). Connexin 43 connexon to gap junction transition is regulated by zonula occludens-1. *Molecular Biology of the Cell*.

[105] Thévenin A. F., Margraf R. A., Fisher C. G., Kells-Andrews R. M., Falk M. M. (2017). Phosphorylation regulates connexin43/ZO-1 binding and release, an important step in gap junction turnover. *Molecular Biology of the Cell*.

[106] Bao H., Yang S., Li H. (2019). The interplay between E-cadherin, connexin 43, and zona occludens 1 in retinal pigment epithelial cells. *Investigative Opthalmology & Visual Science*.

[107] Gonzalez-Casanova J., Schmachtenberg O., Martinez A. D., Sanchez H. A., Harcha P. A., Rojas-Gomez D. (2021). An update on connexin gap junction and hemichannels in diabetic retinopathy. *International Journal of Molecular Sciences*.

[108] Tien T., Barrette K. F., Chronopoulos A., Roy S. (2013). Effects of high glucose-induced Cx43 downregulation on occludin and ZO-1 expression and tight junction barrier function in retinal endothelial cells. *Investigative Opthalmology & Visual Science*.

[109] Trudeau K., Muto T., Roy S. (2012). Downregulation of mitochondrial connexin 43 by high glucose triggers mitochondrial shape change and cytochrome c release in retinal endothelial cells. *Investigative Opthalmology & Visual Science*.

[110] Sankaramoorthy A., Roy S. (2021). High glucose-induced apoptosis is linked to mitochondrial connexin 43 level in RRECs: implications for diabetic retinopathy. *Cells*.

[111] Chen C.-H., Mayo J. N., Gourdie R. G., Johnstone S. R., Isakson B. E., Bearden S. E. (2015). The connexin 43/ZO-1 complex regulates cerebral endothelial F-actin architecture and migration. *American Journal of Physiology—Cell Physiology*.

[112] Sorgen P. L., Duffy H. S., Sahoo P., Coombs W., Delmar M., Spray D. C. (2004). Structural changes in the carboxyl terminus of the gap junction protein connexin43 indicates signaling between binding domains for c-Src and zonula occludens-1. *Journal of Biological Chemistry*.

[113] Toyofuku T., Yabuki M., Otsu K., Kuzuya T., Hori M., Tada M. (1998). Direct association of the gap junction protein connexin-43 with ZO-1 in cardiac myocytes. *Journal of Biological Chemistry*.

[114] Montgomery J., Ghatnekar G. S., Grek C. L., Moyer K. E., Gourdie R. G. (2018). Connexin 43-based therapeutics for dermal wound healing. *International Journal of Molecular Sciences*.

[115] Ghatnekar G. S., ’O’Quinn M. P., Jourdan L. J., Gurjarpadhye A. A., Draughn R. L., Gourdie R. G. (2009). Connexin43 carboxyl-terminal peptides reduce scar progenitor and promote regenerative healing following skin wounding. *Regenerative Medicine*.

[116] Moore K., Bryant Z. J., Ghatnekar G., Singh U. P., Gourdie R. G., Potts J. D. (2013). A synthetic connexin 43 mimetic peptide augments corneal wound healing. *Experimental Eye Research*.

[117] Cheng X., Yang Y.-L., Yang H., Wang Y.-H., Du G.-H. (2018). Kaempferol alleviates LPS-induced neuroinflammation and BBB dysfunction in mice via inhibiting HMGB1 release and down-regulating TLR4/MyD88 pathway. *International Immunopharmacology*.

[118] Zhang J., Yang G.-m., Zhu Y., Peng X.-y., Li T., Liu L.-m. (2015). Role of connexin 43 in vascular hyperpermeability and relationship to rock1-MLC20pathway in septic rats. *American Journal of Physiology—Lung Cellular and Molecular Physiology*.

[119] Hunter A. W., Barker R. J., Zhu C., Gourdie R. G. (2005). Zonula occludens-1 alters connexin43 gap junction size and organization by influencing channel accretion. *Molecular Biology of the Cell*.

[120] Kir D., Schnettler E., Modi S., Ramakrishnan S. (2018). Regulation of angiogenesis by microRNAs in cardiovascular diseases. *Angiogenesis*.

[121] Sun L. L., Li W. D., Lei F. R., Li X. Q. (2018). The regulatory role of micro RNA s in angiogenesis-related diseases. *Journal of Cellular and Molecular Medicine*.

[122] Mukwaya A., Jensen L., Peebo B., Lagali N. (2019). MicroRNAs in the cornea: role and implications for treatment of corneal neovascularization. *Ocular Surface*.

